# Single nucleotide polymorphism (SNP) markers for genetic diversity and population structure study in Ethiopian barley (*Hordeum vulgare* L.) germplasm

**DOI:** 10.1186/s12863-023-01109-6

**Published:** 2023-02-14

**Authors:** Mihret Yirgu, Mulugeta Kebede, Tileye Feyissa, Berhane Lakew, Aemiro Bezabih Woldeyohannes, Mulusew Fikere

**Affiliations:** 1 Department of Applied Biology, School of Applied Natural Science, Adama Science and Technology University, P.O.Box 1888, Adama, Ethiopia; 2 Department of Plant Science, College of Agriculture and Natural Resource, Madda Walabu University, P.O.Box 247, Robe, Oromia Ethiopia; 3 Department of Plant Biology and Biodiversity Management, College of Natural and Computational Sciences, Addis Ababa University, P.O.Box 3434, Addis Ababa, Ethiopia; 4 Institute of Biotechnology, Addis Ababa University, P.O.Box 1176, Addis Ababa, Ethiopia; 5 Holetta Agricultural Research Center, Ethiopian Institute of Agricultural Research, Holetta, Ethiopia; 6 Debre Birhan Agricultural Research Center, Amhara Agricultural Research Institute, Debre Birhan, Ethiopia; 7 Institute of Molecular Bioscience, University of Queensland, Brisbane, Australia

**Keywords:** Accession, Cluster, Diversity, *Ex-situ* conservation, Genetic differentiation

## Abstract

**Background:**

High-density single nucleotide polymorphisms (SNPs) are the most abundant and robust form of genetic variants and hence make highly favorable markers to determine the genetic diversity and relationship, enhancing the selection of breeding materials and the discovery of novel genes associated with economically important traits. In this study, a total of 105 barley genotypes were sampled from various agro-ecologies of Ethiopia and genotyped using 10 K single nucleotide polymorphism (SNP) markers. The refined dataset was used to assess genetic diversity and population structure.

**Results:**

The average gene diversity was 0.253, polymorphism information content (PIC) of 0.216, and minor allelic frequency (MAF) of 0.118 this revealed a high genetic variation in barley genotypes. The genetic differentiation also showed the existence of variations, ranging from 0.019 to 0.117, indicating moderate genetic differentiation between barley populations. Analysis of molecular variance (AMOVA) revealed that 46.43% and 52.85% of the total genetic variation occurred within the accessions and populations, respectively. The heat map, principal components and population structure analysis further confirm the presence of four distinct clusters.

**Conclusions:**

This study confirmed that there is substantial genetic variation among the different barley genotypes. This information is useful in genomics, genetics and barley breeding.

**Supplementary Information:**

The online version contains supplementary material available at 10.1186/s12863-023-01109-6.

## Background

Barley (*Hordeum vulgare* L., 2n = 2x = 14 chromosomes, 5.1 Gb haploid genome size) is one of the main important cereal crops cultivated worldwide in a wide range of environments [1, 2]. Barley has been part of a sustainable food source for humans since pre-historic times. It is mainly used for human food, animal feed, malting and brewing [3, 4]. The crop is a major component of staple food in China, India, Morocco, Ethiopia and Eritrea, the mountainous regions of Bolivia, Ecuador, Colombia and Peru [3].

Barley is the fifth most important cereal crop in Ethiopia after teff, maize, sorghum and wheat both in area of production and amount [5]. Ethiopia is the second-largest barley producer in Africa following Morocco [2], and the average yield of barley in the country is 2.18 tons ha^−1^ [5]. It is grown in various agro-ecologies, ranging from lowland to high altitudes [6, 7] but it performs well at higher altitudes in the northern and central regions of the country [8, 9]. The production of barley in Ethiopia is challenged by abiotic factors (water logging, poor soil fertility, drought and frost) and biotic stress such as net blotch (*Pyrenophora teres*), scald (*Rhynchosporium secalis*) and leaf rust (*Puccinia hordei*) [10, 11]. Understanding, the existence of diverse barley germplasm for yield and yield related traits, resistance against diseases and abiotic stress tolerance, at a molecular level is crucial for barely breeding programs and to enhance its productivity in the country.

Ethiopia is considered as a center of diversity for barley (*Hordeum vulgare* L.), and it is landraces are genetically unique and diverse [12, 13]. Earlier studies have shown that the highest genetic diversity within the Ethiopia barley genetic resource for various useful traits can be valorized for barley breeding [8, 14]. This high diversity is due to diverse agro-ecologies, wide ranges of altitude, soil variability, climate and farming systems and topography together with geographical isolation [15, 16]. Barley's population structure also highly depends on the farming system and altitude in Ethiopia [17].

In the past decades, the Ethiopian Biodiversity Institute (EBI) has collected and conserved more than 16,000 accessions sampled from various agro-ecologies across the country. It is a useful resource for genetic diversity and can play an important role in developing new barley varieties with higher yield potential and other desirable agronomic traits [18]. Konopka [19] stated that the Ethiopian barley collection is one of the world’s ten largest barley collections and is used as a source of elite breeding material for national as well as global breeding programs. It has useful traits such as the source of disease resistance [20], yellow dwarf virus resistance gene [21, 22], powdery mildew resistance [23], barley leaf scald and net blotch [24], high lysine content and protein quality [25], malting and brewing quality [26]. However, most of the early studies were based on either collection from distinct geographical regions only or a few samples collected from wider geographical ranges [27].

Analyzing the molecular diversity and it is genetic relationship encompassed in crop genetic resources is a prerequisite for designing efficient selection in crop breeding programs and for developing conservation and valorization strategies. Previous studies have used DNA markers to determine the genetic diversity of barley. Markers such as amplified fragment length polymorphism (AFLP) [28], restriction fragment length polymorphisms (RFLPs) [29], and simple sequence repeats (SSR) [27, 30–33] have been employed and provided crucial information for barely breeding programs.

Currently, single nucleotide polymorphism (SNP) markers are the markers of choice for genetic diversity studies [34], genome-wide association mapping [35, 36], genomic selection [37], phylogenetic relationships and population evolutionary history studies [38, 39]. SNPs are the most abundant and robust, feasible for automated high-throughput genotyping [40], highly reproducible and can be used to identify variants [41], replacing the earlier markers due to high throughput, efficiency and cost-effectiveness [12].

A recent study by Teklemariam et al. [42] has observed a weak correlation between geographic distance and genetic differentiation of some Ethiopian barely germplasm collections using SNP markers. However, the genetic diversity of Ethiopia's diverse barley germplasm collection has not been adequately characterized and exploited using advanced tools such as SNP markers. Therefore, this study was designed to assess the genetic diversity and population structure of Ethiopian barley genotypes using SNP markers.

## Results

### SNP variation and markers distribution

**Fig. 1:**
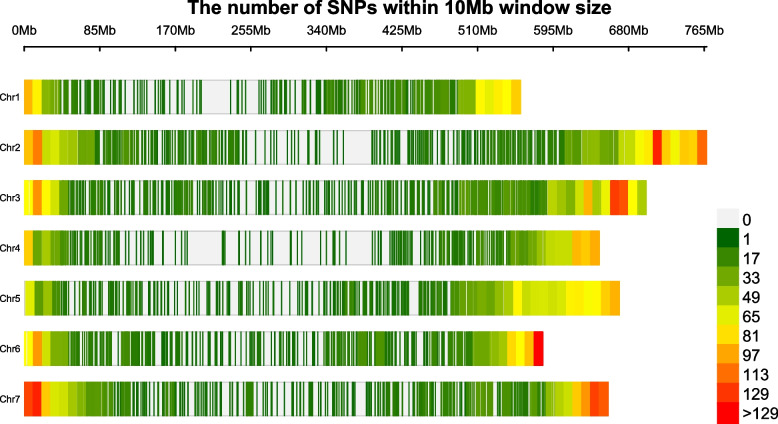
Distribution of SNP markers within 10 Mb window size across seven chromosomes. Colored bars are SNP counts in 10 Mb interval

The single nucleotide polymorphism (SNP) distribution of the 10,103 SNPs remaining after imposing a quality control threshold was plotted in 10 Mb (megabase pair) window size across the *H.vulgare* genome (Fig. 1). Variant distribution was not completely uniform across the chromosomes. We detected an average of 97 SNPs per 10 Mb. The highest SNP density (> 129 SNPs/10 Mb) was observed on chromosomes Chr2, Chr3 and Chr6 and Chr7. The lowest average SNP density was found on chromosome Chr1 (< 33 SNPs/10 Mb).

### Genetic diversity and relationship

The genetic parameters included gene diversity, heterozygosity (the ratio of observed to expected heterozygosity), minor allelic frequency (MAF) and the polymorphic information content (PIC) of the 10,103 SNP markers of 105 barley genotypes were presented in Table 1. The result showed that the average genetic diversity was 0.253 and ranged from 0.177 to 0.311. The highest gene diversity (0.311) was observed for genotypes collected from the Amhara region followed by the Oromia (0.288), SNNP (0.266), Tigray (0.221) and ICARDA (0.177). The PIC values ranged from 0.151(ICARDA) to 0.271(SNNP) with an average polymorphism value of 0.216, which indicates high genetic diversity between the barley genotypes. The MAF ranged from 0.092 to 0.146, with an average of 0.118. Genotypes from the Oromia region showed the highest MAF (0.146) followed by ICARDA (0.145) collection and Amhara (0.107) genotypes. However, the lowest MAF was exhibited in the SNNP (0.101) and Tigray (0.092) genotypes. In addition, the observed heterozygosity (Ho) ranged from 0.015 in the Tigray to 0.088 in the Oromia genotypes with an average of 0.045. On the other hand, the expected heterozygosity (He) was higher than that observed heterozygosity (Ho), ranging from 0.133 to 0.215 with an average of 0.163.

**Table 1:** Gene diversity, observed heterozygosity (Ho), expected heterozygosity (He), minor allele frequency (MAF) and the polymorphism information content (PIC) of the 105 barley genotypes

Regions	Genotypes	GD	Ho	He	MAF	PIC
Amhara	31	0.311	0.071	0.158	0.107	0.195
Oromia	32	0.288	0.088	0.215	0.146	0.211
SNNP	21	0.266	0.027	0.145	0.101	0.271
Tigray	19	0.221	0.015	0.133	0.092	0.250
ICARDA/Other	2	0.177	0.022	0.164	0.145	0.151
**Average values**	**105**	**0.253**	**0.045**	**0.163**	**0.118**	**0.216**

*Abbreviations*: *GD* Gene diversity, *Ho* Observed heterozygosity, *He* Expected heterozygosity, *MAF* Minor allele frequency, *PIC* Polymorphism information content, *SNNP* Southern Nations,Nationalities and Peoples, *ICARDA* International Centre for Agricultural Research in the Dry Areas

### Analysis of molecular variance (AMOVA)

The analysis of molecular variance (AMOVA) showed that the proportion of variance within the barley populations was significantly higher (52.85%, *P* < 0.0001) than the variation within accessions (46.43%, *P* < 0.0001) (Table 2). Conversely, a significantly lower level of genetic variation (0.72%,* P* < 0.0001) was recorded between the barley populations (Table 2).

**Table 2:** Analysis of molecular variance (AMOVA) of 105 barley population based on SNP markers

**Source of variation**	**Mean square**	**Estimated variance**	**Proportion of Variance (%)**	**Probability** (***p***) **value**
Between populations	2349.75	10.8	0.72	***
Within populations	1742.11	820.77	52.85	***
Within accessions	475.33	582.41	46.43	***
**Total**	**1588.53**	**1413.98**	**100**	

Significant codes: 0 ‘***’ 0.001, ‘**’ 0.01, ‘*’ 0.05, ‘ns’ 0.1, ‘ns’ 1, ns-non significant

### Genetic differentiation

Pairwise genetic differentiation (F_ST_) among barley populations was computed using SNP markers (Table 3). The lower values of 0.019, 0.022, and 0.038 genetic differentiations were recorded between the barley genotypes originating from Amhara and Oromia, Amhara and SNNP, and SNNP and Oromia regions, respectively (Table 3). Although moderate genetic differentiation was recorded between the Tigray and SNNP (0.117) followed by Tigray and Amhara (0.089) and Tigray and Oromia (0.088) (Table 3). This revealed that the barley genotypes obtained from those regions of origin the existence in the highest genetic variations and distant relationships.

**Table 3:** Pairwise genetic differentiation (F_ST_) among barley populations using SNP markers

Regions	Amhara	SNNP	Oromia
Amhara	-	-	-
SNNP	0.022	-	-
Oromia	0.019	0.038	-
Tigray	0.089	0.117	0.088

*Abbreviation*: *SNNP* Southern Nations, Nationalities and Peoples

### Genetic distance and identity

A high genetic distance (0.08) was recorded among barley genotypes obtained from Tigray and ICARDA, which indicates the existence of high genetic differences among the genotypes (Table 4). The genetic distance ranged from 0.002 to 0.08. Besides, the genetic identity ranged from 0.971 to 0.998, and the highest genetic identity was found among Amhara and Tigray, Amhara and ICARDA, and Oromia and SNNP. However, a lower (0.971) genetic identity value was observed between genotypes originating from SNNP and ICARDA (Table 4).

**Table 4:** Genetic distance (below diagonal) and genetic identity (above diagonal) between 105 barley genotypes based on SNP markers

Regions	Amhara	Oromia	SNNP	Tigray	ICARDA/Other
Amhara		0.991	0.993	0.998	0.998
Oromia	0.009		0.998	0.990	0.990
SNNP	0.007	0.012		0.991	0.971
Tigray	0.012	0.021	0.004		0.992
ICARDA/Other	0.002	0.011	0.031	0.08	

*Abbreviations*: *SNNP* Southern Nations, Nationalities and Peoples, *ICARDA* International Centre for Agricultural Research in the Dry Areas

### Principal component analysis

**Fig. 2:**
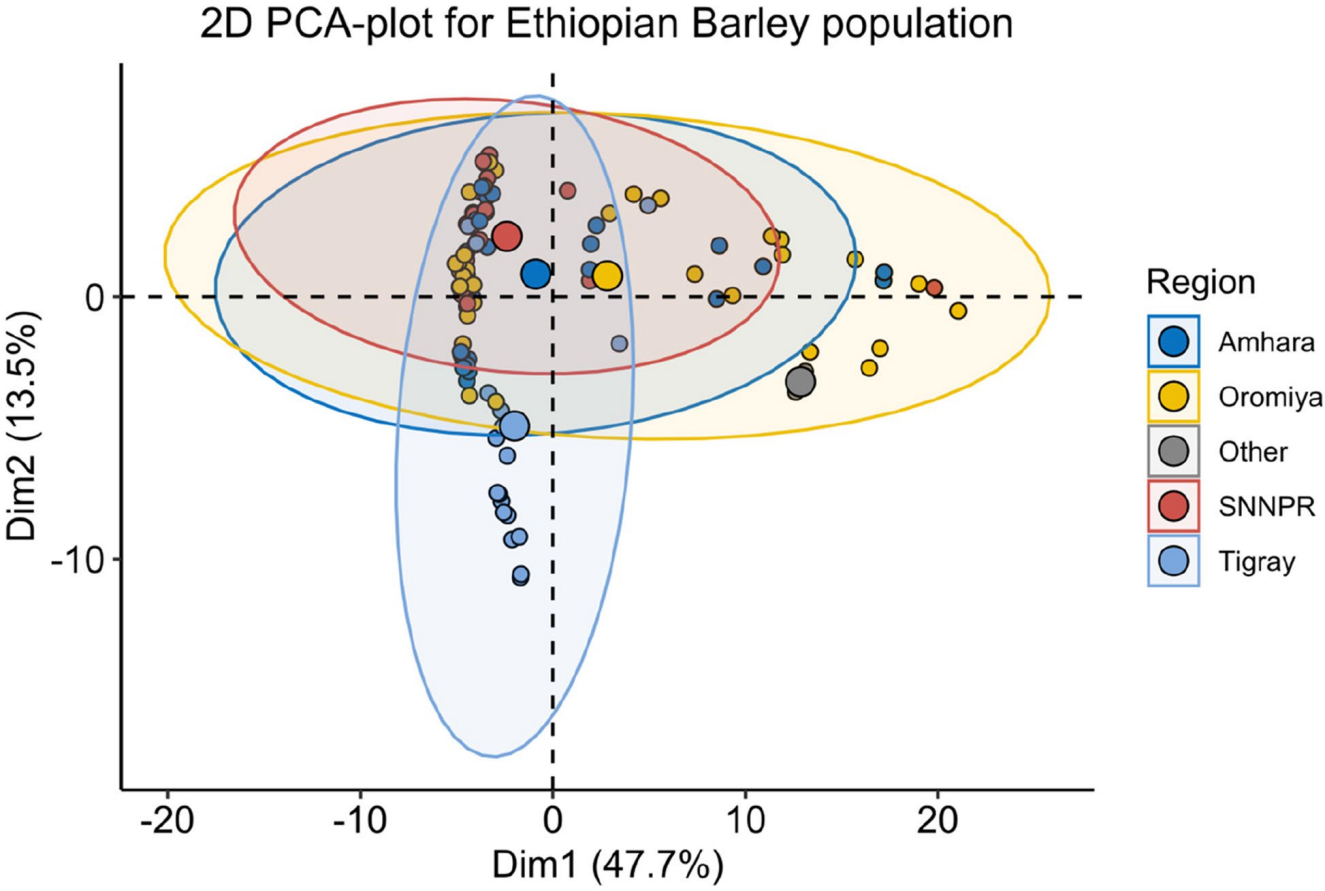
Principal component analysis showing the clustering among the region of origins of 105 barley genotypes using 10,103 SNP markers. The genotypes are grouped into three main groups. Colored dots are genotypes from various region of origins and grouped by regions, according to the legend. PC1 and PC2 are shown on the X and Y-axis, respectively, aside from their explained variance. Abbreviations: SNNPR = Southern Nations, Nationalities and Peoples Region

**Fig. 3:**
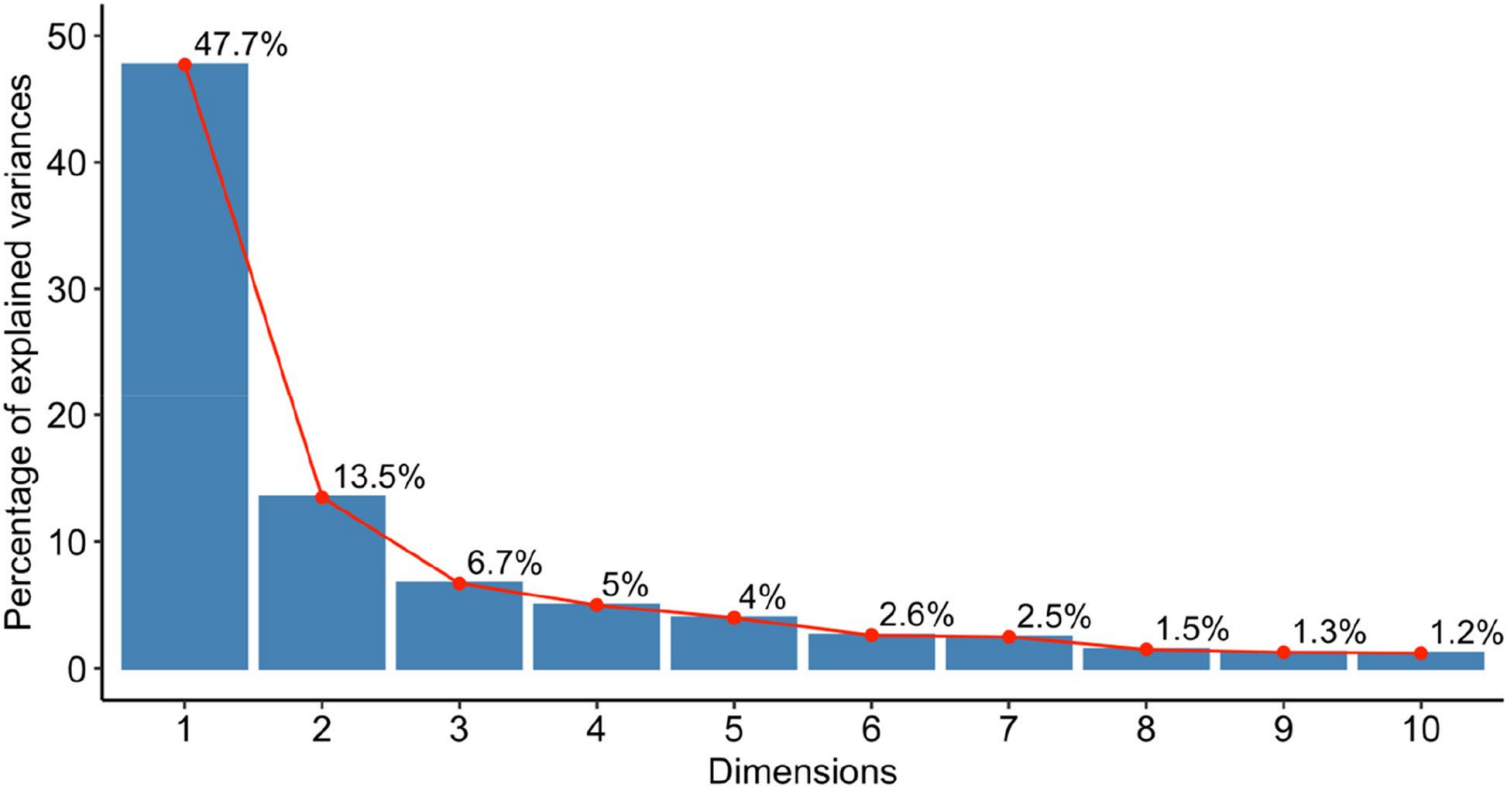
Percentages of explained variances for 105 barley genotypes using SNP markers

To quantify the genetic variation between genotypes, we perform a principal component analysis (PCA). Four clusters were observed which accounted for 47.7% and 13.5% of the total variation in PC1 and PC2 of the total variance, respectively (Fig. 2). Oromia and Amhara genotypes were loadings in PC1 whereas Tigray and SNNP regions were the major loadings to PC2 (Fig. 2). The lowest total variance was obtained for PC3 (6.7%) and PC4 (5%) (Fig. 3). As shown in Fig. 3, the third and consecutive PCs resulted in a lower percentage of contribution to the total genetic variance. The PCA groups showed slightly unclear-cut separation due to an explicit genetic relatedness between the Amhara and SNNP, and Oromia and Amhara genotypes. The first group assembled Amhara and SNNP genotypes. The second group consisted of Tigray genotypes, and the last group contained the remaining genotypes from Oromia found in all groups (Fig. 2).

### Genetic relatedness

**Fig. 4:**
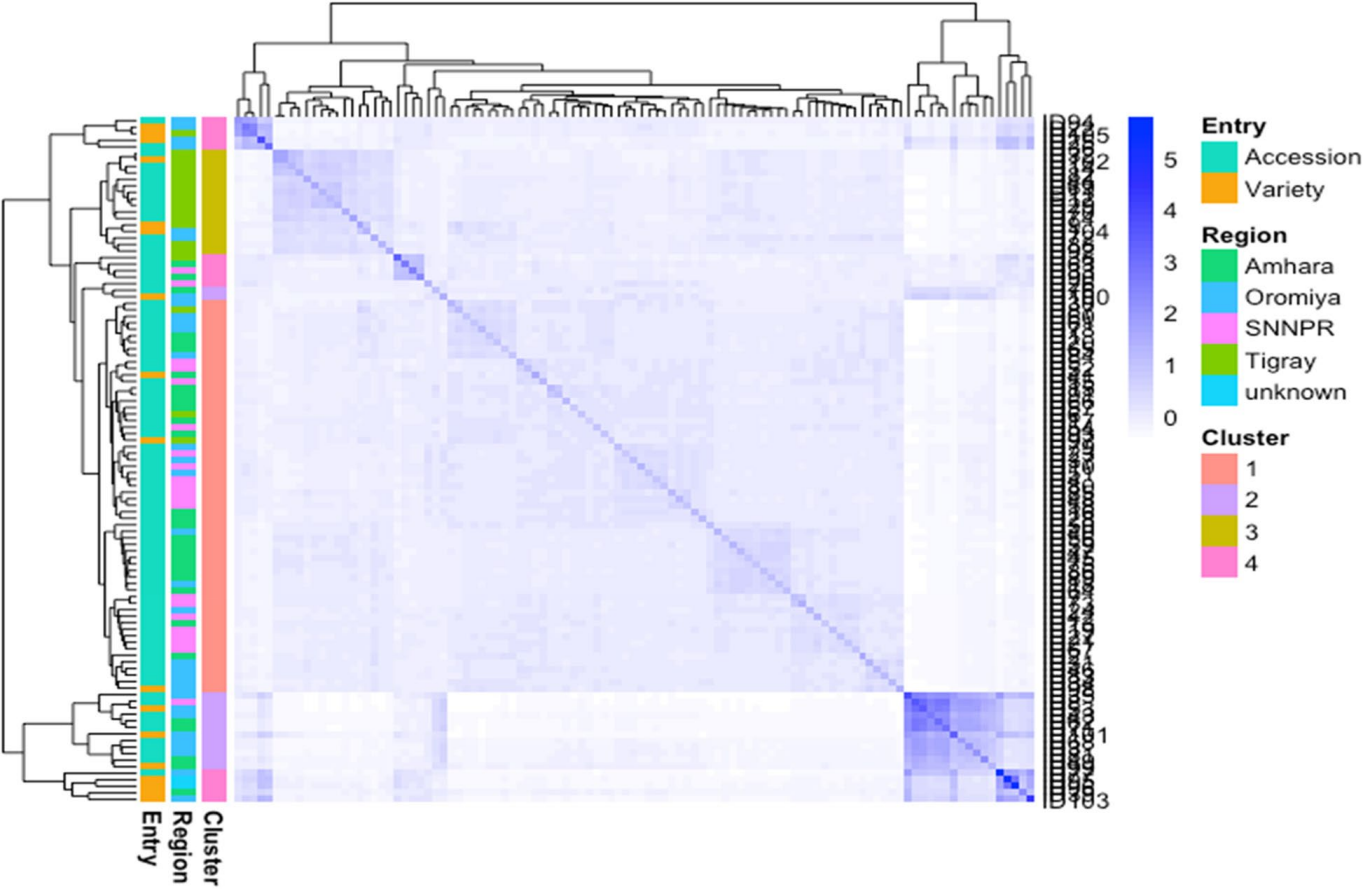
Heat map analysis shows relationship distance based on the color distribution among 105 barley populations genotyped using 10,103 SNP markers (for genotypes name and collection origins refer to Additional file 1: Table S1). Colored plots on the left side are entry genotypes, regions and clusters at the various stages, according to the legend to the right. On the left of the matrix, barley genotypes are grouped into four distinct clusters. Abbreviations: SNNPR = Southern Nations, Nationalities and Peoples Region, Unknown = unidentified genotype names

We perform genetic relatedness analysis using the refined SNP dataset of 105 genotypes. The heat map was used to visualize the genetic relatedness across the population. The heat map plot depicted four clusters (Fig. 4, Table 5). Cluster I contained the largest number of barley genotypes (*n* = 60) followed by cluster III (*n* = 16), cluster IV (*n* = 15) and cluster II (*n* = 14). The number of genotypes belonging to distinctive clusters varies from 14 in Clusters II to 60 in Cluster I (Fig. 4, Table 5). Of all, cluster I was the largest cluster, consisting of 3(2.9%), 16(15.4%), 18(17.2%) and 23(21.9%) from Tigray, Oromia, SNNP and Amhara regions, respectively. In terms of altitude based clustering, the majority of the genotypes were included at an altitude of 2001–3000 masl. However, cluster II consists of a small number of barley genotypes, commonly from Amhara (4.8%) and Oromia (7.6%) regions with originated at medium and high altitude ranges (Fig. 4, Table 5). Cluster III encompassed genotypes only from Oromia (1.9%) and Tigray (13.3%) regions. The greatest number of genotypes included from an altitude of 2001–2500 masl. Cluster IV comprises genotypes from all regions with the highest percentage in the Oromia (5.7%) and the genotypes comprised at the medium altitude (Fig. 4, Table 5).

**Table 5:** Clustering of 105 barley genotypes into four groups using SNP markers, the distribution of genotypes across the region of origins and altitudinal ranges

Regions	Cluster I	Cluster II	Cluster III	Cluster IV	Total
Oromia	16	8	2	6	32
Amhara	23	5	0	3	31
Tigray	3	0	14	2	19
SNNP	18	1	0	2	21
ICARDA/Other	0	0	0	2	2
**Total**	**60**	**14**	**16**	**15**	**105**
**Altitude Ranges**					
< 2000	8	1	2	3	14
2001–2500	22	5	9	8	44
2501–3000	21	3	5	3	32
> 3001	9	5	0	1	15
**Total**	**60**	**14**	**16**	**15**	**105**

*Abbreviations*: *SNNP* Southern Nations, Nationalities and Peoples, *ICARDA* International Centre for Agricultural Research in the Dry Areas

### Population structure

**Fig. 5:**
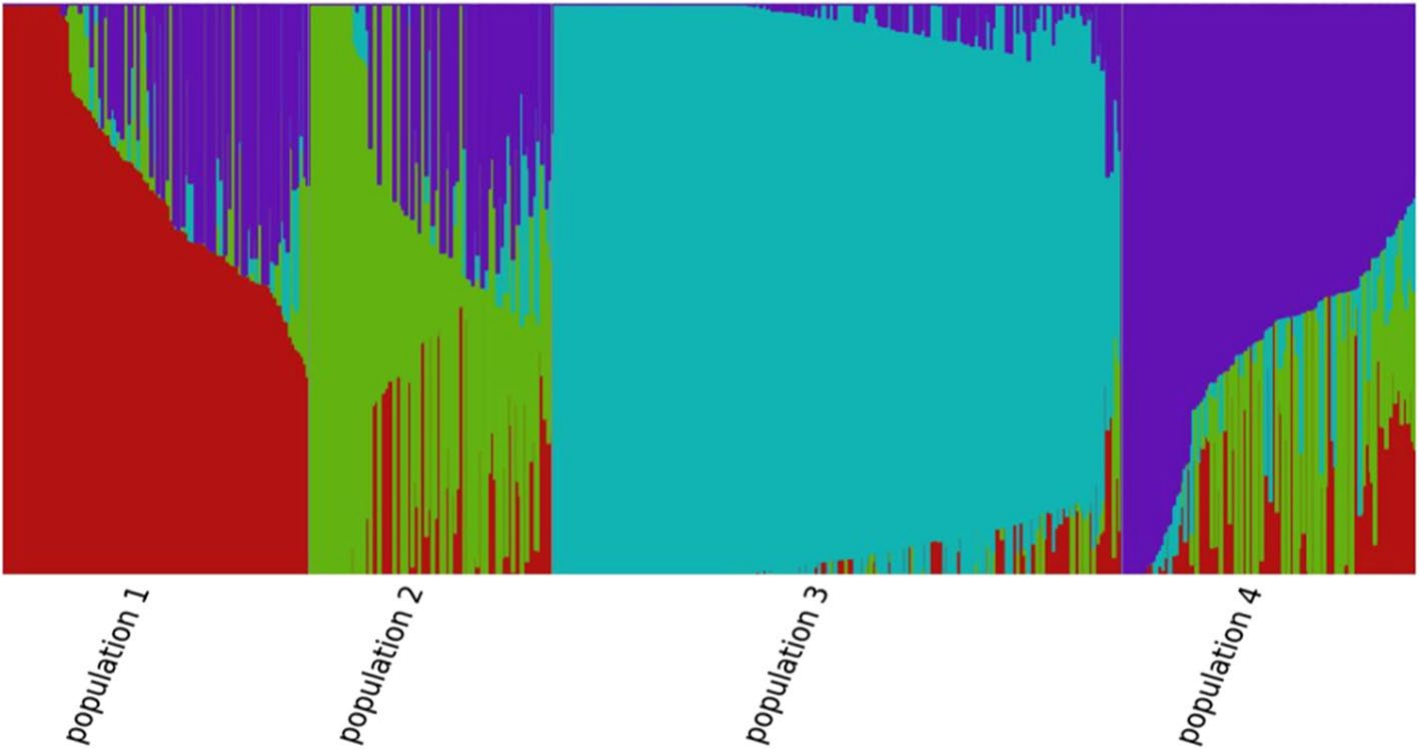
Population structure of 105 barley genotypes (K = 4). Each vertical bar represents genotypes that are divided into K-colored segments

A total of 10,103 SNP markers were used for the population structure analysis of the 105 barley genotypes. The best number of K, which clearly defined the number of populations as K = 4, revealed that four subpopulations should include all the 105 barley genotypes with a great probability (Fig. 5). Each K is shown in diverse colors (Fig. 5), the subpopulations described in blue-green and purple observed a high proportion of the variation with the barley genotypes confirming the results of AMOVA and heat map results.

## Discussions

The genetic diversity study of germplasm is the greatest way to understand ancestry relations and to achieve proficient management of crop genetic resources to improve breeding programs [43, 44]. Such genetic diversity analysis was essential for plant breeders to perform strategic integration and target selection while preserving important economic traits of individual crops. In this study, the highest gene diversity (GD = 0.311) was revealed in barley genotypes (Table 1). This high gene diversity may be a natural crossing due to cultivating mixed genotypes in a field [45]. The average gene diversity was 0.253, which is lower than that indicated in worldwide barley genotypes (GD = 0.388) [46], in ICARDA spring barley collections (GD = 0.366) [47], in Nordic spring barley collections (GD = 0.359) [48] and Egyptian barley collections (GD = 0.550) [49]. The PIC ranged was 0.151 to 0.271 with an average polymorphism value of 0.216 (Table 1). A previous study indicated that PIC values > 0.5 mean highly informative markers, 0.5 > PIC > 0.25 is an informative marker, and PIC < 0.25 is a fairly informative marker [50]. Therefore, our results showed that the SNP markers were informative and polymorphic. The average and range values of polymorphism observed in the present study were lower than the reported range values of 0.474 to 0.652 with an average value of 0.552 in barley landraces [33], 0.34 to 0.83 with an average of 0.57 in barley collections [51]. Another study with Ethiopian durum wheat by Alemu et al. [52] estimated the ranged value from 0.01 to 0. 375 with a mean PIC of 0.375. Higher MAF (MAF = 0.146) with an average value of 0.118 was found in barley genotypes (Table 1), indicating further valuable genes can be exploited from those genotypes. The lower MAF obtained may be due to the lower number of genotypes studied from those regions. Our result is lower than that reported for the MAF range was 0.517 to 0.520 in Ethiopian sorghum with a mean value of 0. 518 [53].

The observed heterozygosity was higher (Ho = 0.088) in the Oromia region, indicating the existence of high genetic variability between the genotypes (Table 1). This may be due to the high rate of outcrossing in barley [54]. In this work, the average observed heterozygosity was smaller (Ho = 0.045) than the average expected heterozygosity (He = 0.163), showing highly related among the barley genotypes. This could be related to the existence of gene flow along with the regions each growing season during the exchange of seeds and interbreeding. Comparatively overall lower heterozygosity is obtained in all the regions, this may be due to the factor explained by the cleistogamy in barley that, the flower sheds its pollen before opening, forcing plants with this habit to be entirely autogamous, which can reduce the heterozygosity [55]. Our result is lower than the reported ranges observed from 0.594 to 0.662, and the expected 0.688 to 0.773 heterozygosity for Ethiopian barley landraces [33]. This is probably due to the source of genotypes and the samples included improved varieties, which are obtained in the lower heterozygosity ranges in our study.

A higher genetic variation of 52.85% was observed within the populations, showing the variations among the barley genotypes in the regions (Table 2). This confirmed that Ethiopia has been recognized as a center of diversity for barley [14]. However, in our study, the genetic diversity of barley genotypes was 0.253, which was lower than (60.31%) for the North African germplasm [56]. The existence of variations in our work may be because of the outcrossing that leads to introducing new genetic components into the population. The large genetic diversity within populations could be a result of natural adaptation or extensive exchange of seeds among farmers between environments [57]. Therefore, the presence of genetic variation within the populations obtained in this study can be utilized in breeding programs to enhance barley productivity, the parental line selection from within the populations could be more valuable compared to selection from among the populations. The AMOVA also showed the genetic variation (46.43%) within accessions (Table 2), this could be associated with climatic variability and agro-ecological heterogeneity in the region of origins. The presence of diversity within accessions confirmed the potential of a genetic variant, which is the source material for barley breeding [30, 33]. The utilization of diversity within accessions using pure-bred selection has been confirmed to provide germplasm for desirable traits [8]. The presence of genetic variation within the populations and accessions suggests that there might be a margin for barley improvement in genomic selection for cultivar development.

The genetic variance fixation index (F_ST_) was an evaluation of population variations because of genetic structure [33, 58]. In the present study, lower values of (F_ST_ = 0.019, 0.022, 0.038) genetic differentiations were shown in Table 3. This showed that the barley genotypes originating from those regions of origin had very close ancestry, and the close origins may be due to high seed exchange that can help barley breeding to exploit hybrids. This work obtained that the very closest pairwise value (F_ST_ = 0.019) was observed between the Amhara and Oromia genotypes while distant dissimilarity (F_ST_ = 0.117) between the SNNP and Tigray genotypes (Table 3). Tigray genotypes were most distantly (F_ST_ = 0.089–0.117) related to other regions of the populations, which showed geographical origins could be the basis of a genetic variant. The genotypes obtained from the Tigray region may be exploited as a source of breeding materials to improve genetic diversity in barley breeding through hybridization programs. Our study result revealed moderate genetic differentiation (F_ST_ = 0.117) for the pairwise comparison among the barley population implying a smaller amount of genetic relatedness and this indicated a lesser genetic relationship. This result was confirmed by genetic differentiation largely higher in marginal populations than in the favorable environment. Marginal stands are grouped by geographic and genetic separation because of spatial segregation and restricted gene flow [59]. A similar finding was reported in this study by Dido et al. [33] indicating moderate genetic differentiation (F_ST_ = 0.082) was shown in the Ethiopian barley population. On the contrary, the largest genetic differentiation (F_ST_ = 0.257) was revealed between the barley population by Allel et al. [56].

Genetic distance is the measure of genetic differences that exist among individuals or populations and can be measured by allelic differences [60]. The present study showed the highest genetic distance (0.08) was found between genotypes derived from Tigray and ICARDA (Table 4), which indicated that the barley genotypes had a high genetic difference. This could be due to the restricted gene flow and the influence of eco-geographical differences on the existence of high genetic distance. On the other hand, the least genetic distance (0.002) was recorded between Amhara and ICARDA (Table 4). Research report explained that the lower genetic distance among collection regions probably has high levels of the farmer to farmer seed exchange and gene flow across regions [53].

The genetic identity ranged from 0.971 to 0.998 (Table 4). The least genetic identity was observed between genotypes collected from SNNP and ICARDA. However, the highest genetic identity (0.998) was from Amhara and Tigray, Oromia and SNNP, and Amhara and ICARDA (Table 4). This is possibly due to natural selection for shared genetic components being the key force in determining the high genetic identity between the geographical origins. Genetic divergence as the lower cause is a result of geographical isolation and distinctive agro-ecological conditions shared by genetic materials [61]. Our result is relatively similar to the genetic identity of North African barley germplasm (0.956) reported in an earlier study by Allel et al. [56].

The first two PCA were explaining 61.2% of the total genetic variation (Fig. 2). This indication highlights the potential of highly informative and selective SNP markers for genetic studies in barley, which might underpin conservation and future breeding efforts. Besides, this result showed genetic differences exist among genotypes confirming the result indicated by AMOVA, which revealed a significant genetic variation within population and accessions. There was a comparison to the present study that has been reported in earlier studies on barley germplasm [49, 51], durum wheat [52] and sorghum germplasm [53, 62]. The heat map analysis also grouped the genotypes into four major clusters, reflecting the origin of the genotypes and their genetic relationships (Fig. 4, Table 5). Likewise, the North African region of barley collection from 14 countries was grouped into four clusters [51]. Our result is also parallel with clustering the Ethiopian barley landraces into three clusters [33, 42] and the Egyptian barley genotypes into three main groups using SNP markers [49].

The present result has shown that the distribution pattern of the genotypes into different groups indicated the existence of significant genetic variations among the barley genotypes. For example, among the clusters, cluster I was the largest cluster containing 60 genotypes (57.2%), and most of the genotypes included an altitude of 2001–3000 masl (Fig. 4, Table 5). These stated that structure analysis grouped the barley genotypes with greater genotypic similarity and this may be used as a source of breeding material to enhance genetic variants in barley breeding. Clustering genotypes into genotypically similar clusters of diverse collections are significant for barley improvements such as selecting parents for hybrid [49, 63] and the development of modern breeding lines [64]. In the genotypes, groups were admixed into the varied clusters irrespective of their collections origins (Fig. 4, Table 5). For instance, genotypes collected from the Oromia region (32) were grouped into four clusters (sixteen genotypes in cluster I, eight genotypes in cluster II, two genotypes in cluster III and six genotypes in cluster IV). Genotypes (31) were collected from the Amhara region also clustered into three groups; 23 genotypes in cluster I, 5 genotypes in cluster II and 3 genotypes in cluster IV (Fig. 4, Table 5). This finding agrees with the admixture that could be associated with gene flow facilitated by the continuous exchange of seeds among smallholder farmers in various agro-ecologies in the shared market and the contentious introduction of new seeds into the respective growing regions [33, 52, 65, 66]. The analysis of population structure also confirms the barley genotypes clustered into four subgroups (Fig. 5), while they showed no clear clustering pattern of grouping. These unstructured grouping and the mixed genetic background suggested that the genotype shared a similar lineage, this is possibly due to the high exchange of planting material between the region of origins. A similar result was reported by the absence of consistent population structure among the Ethiopian barley landraces [31, 42] and Jordan barley germplasm [67], which was related to high seed-mediated gene flow.

## Conclusions

In the current study, the SNP data generated using advanced molecular tools provide useful information that can be utilized in breeding and genetic research in barley. Based on SNP data, the barley genotypes were genetically divergent. The AMOVA indicated high genetic variations within the populations and genotypes. This high diversity could be the foundation for developing and generating desirable new barley varieties with superior grain yield potential and wide adaptability, enhanced with abiotic and biotic resistant traits. This study identified four subpopulations, considered as four independent subpopulations in the improvement program, but was not grouped the genotypes according to collection origin and adaption zone. This information on genetic diversity and population structure of Ethiopian barley genotypes will be applied for current and future research using genome-wide association and genomic selection for economically useful traits in barley.

## Methods

### Plant materials

One hundred five barely panels comprised of eighty-five barley accessions and sixteen improved varieties including two landraces and two wild crosses were used in this study. These lines were chosen based on cultivars' regional passport information and the breeding merit they have for subsequent germplasm enhancement. The eighty-five barley accessions were obtained from the *ex-situ* collection of the Ethiopian Biodiversity Institute (EBI) along with their passport data (Additional file 1: Table S1). The random sampling procedure was modified to allow the equal representation of barley accessions from the 1976 to 2018 collection periods in four regions of Ethiopia (Oromia, Amhara, Tigray, and Southern Nations, Nationalities and Peoples) (Additional file 2: Fig. S1). To get detailed information on genetic diversity, genotypes were collected from a wide range of altitudes (≤ 2000, 2001–2500, 2501–3000 and ≥ 3001 masl) (Additional file 2: Fig. S1). The improved varieties including landraces were obtained from Universities, national and regional agricultural research centers, and two wild crosses of barley from Debre Zeit Agricultural Research Center (DZARC) primarily introduced from the International Centre for Agricultural Research in the Dry Areas (ICARDA) (Additional file 1: Table S1).

### Planting, leaf sampling and genomic DNA extraction

Each barley genotype was sowed on 02 August 2021 in a seedling tray, five seedlings each, in a greenhouse at National Agricultural Biotechnology Research Center (NABRC), Holetta. For two weeks old barley leaf samples were collected and pooled from the same genotype in equal amounts. Then samples were placed into 96 collection plates (96-well plate holds 12 × 8-strip tubes). During the sample preparation, the leafcutters (scissors) were sterilized with 70% alcohol before cutting the next genotype to prevent cross-contaminate. The collected leaf samples were first freeze-dried at -20 °C for 24 h. Then the freeze-dried leaf samples were shipped for genotyping to the Integrated Genotyping Service and Support (IGSS) platform located at Biosciences Eastern and Central Africa- International Livestock Research Institute Hub (BecA-ILRI Hub) based in Nairobi. Genomic DNA from 105 barley genotypes was extracted using the Nucleomag Plant Genomic DNA extraction kit following the manufacturer's instructions. The genomic DNA concentration was used in the range of 50–100 ng/μl. The quality and quantity of extracted DNA in each sample were determined by electrophoresis on 0.8% agarose gel.

### SNP genotyping

Genomic libraries were constructed according to Kilian et al. [68], complexity reduction method through digestion of genomic DNA using a combination of PstI and HpaII enzymes and ligation of bar-coded adapters followed by PCR amplification of adapter-ligated fragments. Libraries were sequenced using single-read sequencing runs for 77 bases. Next-generation sequencing technology was carried out using HiSeq2500 (Illumina). The markers scoring was achieved using DArTsoft14, which is an in-house marker scoring pipeline based on algorithms. Two types of markers were scored, SilicoDArT markers and SNP markers which were both scored ‘1’ for presence, and ‘0’ for absence and ‘-’ for calls with non-zero count however too low counts to scored confidently as “1” for the SilicoDArT while the sequences SNPs were scored ‘0’ for reference allele homozygote, ‘1’ for SNP allele homozygote and ‘2’ for heterozygote. Totalities of 31,646 silicoDArTs and SNP markers were employed to genotype the materials. Both SilicoDArT and SNP markers were aligned to the reference genome of *Hordeum vulgare*_v2.0 [69], to identify chromosome positions. Our study which included only SNP markers data was used for this analysis after SNP calling and imputation.

### SNP calling and data filtering

An initial set of 14,454 single nucleotide polymorphisms (SNPs) containing monomorphic and undefined chromosomes or positions were removed from the raw dataset. Then further quality control was performed, and SNP markers with > 95% call rate, minor allele frequency (> 5%), and missing rate per sample (< 10%) and per SNP (< 30%) were retained for downstream analysis using R software version 4.1.3 [70]. The diversity analysis was performed using complete (non-missing) data; therefore, SNP with missing loci was imputed using the R package snpReady [71]. Finally, 10,103 (69.90%) SNP markers were retained for further analysis.

### Data analysis

Genetic parameters such as polymorphism information content (PIC), genetic diversity, heterozygosity (observed and expected), minor allele frequency (MAF), genetic differentiation and genetic distance were computed using the R package snpReady [71]. The molecular variance analysis (AMOVA) was performed at different hierarchical levels (among and within the populations and accessions) in the stats R package using the *aov* function.

To explore the genetic structure of the 105 barley accessions we first undertook a cluster analysis using the agglomerative hierarchical algorithm (wards method, Euclidean distance) (Anderberg [72] of the locus-by-entry reference allele count table for the SNP markers that passed the filtration process) and in the dendrogram was plotted across the testing location or regions. The elbow method (implemented in the R package factoextra) was used to determine the optimum number of clusters (k) [73, 74]. The best *K*-value for estimating an optimum subpopulation size for the dataset was determined based on peak Δ*K* values following the Evanno method [75]. A principal component analysis (PCA) of the SNP markers was conducted using the *prcomp* function to summarise the contributions of each part to the variation that existed in the population. The genetic diversity pattern among the population was visualized using the *pheatmap* R package [71].

## Supplementary Information

**Additional file 1: Table S1. **List of 105 barley genotypes obtained from the *ex-situ* collection of the Ethiopian Biodiversity Institute (EBI), Ethiopian Institute of Agricultural Research (EIAR) and Universities with the region of origins and altitude ranges.

**Additional file 2: Figure S1.** The administrative map of Ethiopia indicates the collection points of the barley genotypes. Dots represent the barley genotypes in different colors depending on various region of origins and altitude ranges, according to the legend.

## Data Availability

The datasets generated and/or analyzed during the current study are available in the European Variation Archive (EVA) at EMBL-EBI under accession number PRJEB59022 (https://www.ebi.ac.uk/eva/?eva-study=PRJEB59022).

## Abbreviations

EBI: Ethiopian Biodiversity Institute
EIAR: Ethiopian Institute of Agricultural Research
DZARC: Debre Zeit Agricultural Research Center
ICARDA: International Centre for Agricultural Research in the Dry Areas
SNNP: Southern Nations, Nationalities and Peoples
NABRC: National Agricultural Biotechnology Research
AMOVA: Analysis of molecular variance
IGSS: Integrated Genotyping Service and Support
BecA-ILRI Hub: Biosciences Eastern and Central Africa- International Livestock Research Institute Hub
