# Supplementary material for: Single nucleotide polymorphism (SNP) markers for genetic diversity and population structure study in Ethiopian barley (Hordeum vulgare L.) germplasm
Source: BMC Genom Data. 2023 Feb 14;24:7. doi: 10.1186/s12863-023-01109-6 (PMC9930229; doi:10.1186/s12863-023-01109-6)
Supplement: Supplementary file 1 — Additional file 1: Table S1. List of 105 barley genotypes obtained from the ex-situ collection of the Ethiopian Biodiversity Institute (EBI), Ethiopian Institute of Agricultural Research (EIAR) and Universities with the region of origins and altitude ranges. [file 12863_2023_1109_MOESM1_ESM.docx]

**Supplementary Files**

**Additional file 1: Table S1.** List of 105 barley genotypes obtained from the *ex-situ* collection of the Ethiopian Biodiversity Institute (EBI), Ethiopian Institute of Agricultural Research (EIAR) and Universities with the region of origins and altitude ranges.

| ID | **Genotypes** | **Regions** | **Altitudes** | ID | **Genotypes** | **Regions** | **Altitudes** | ID | **Genotypes** | **Regions** | **Altitudes** | ID | **Genotypes** | **Regions** | **Altitudes** |
| --- | --- | --- | --- | --- | --- | --- | --- | --- | --- | --- | --- | --- | --- | --- | --- |
| 1 | 235057 | 2 | 3 | 28 | 243605 | 2 | 3 | 55 | 208845 | 4 | 3 | 82 | 15255 | 3 | 2 |
| 2 | 3509 | 2 | 3 | 29 | 242584 | 3 | 1 | 56 | 3470 | 2 | 1 | 83 | 239538 | 1 | 2 |
| 3 | 237838 | 1 | 4 | 30 | 18319 | 1 | 2 | 57 | 1625 | 2 | 3 | 84 | 29523 | 1 | 3 |
| 4 | 235530 | 4 | 4 | 31 | 18871 | 1 | 3 | 58 | 16739 | 4 | 4 | 85 | 16857 | 4 | 3 |
| 5 | 208843 | 4 | 3 | 32 | 235252 | 3 | 2 | 59 | 24237 | 2 | 1 | 86 | 3473 | 2 | 2 |
| 6 | 242092 | 3 | 3 | 33 | 244772 | 4 | 3 | 60 | 3611 | 1 | 1 | 87 | 238349 | 3 | 3 |
| 7 | 16804 | 4 | 2 | 34 | 15366 | 1 | 2 | 61 | 29524 | 1 | 2 | 88 | 242094 | 3 | 2 |
| 8 | 27503 | 4 | 1 | 35 | 208857 | 1 | 2 | 62 | 236811 | 2 | 4 | 89 | 17255 | 2 | 3 |
| 9 | Wolelay | 3 | 2 | 36 | 15397 | 1 | 2 | 63 | 9950 | 2 | 2 | 90 | 243598 | 2 | 4 |
| 10 | 16853 | 4 | 3 | 37 | 208815 | 1 | 3 | 64 | 26519 | 2 | 2 | 91 | 1652 | 2 | 4 |
| 11 | 234311 | 3 | 2 | 38 | 25919 | 3 | 2 | 65 | 15291 | 2 | 4 | 92 | 15277 | 3 | 3 |
| 12 | 242583 | 3 | 1 | 39 | Yedogit | 2 | 1 | 66 | 24126 | 2 | 4 | 93 | 1661 | 2 | 4 |
| 13 | 236254 | 2 | 2 | 40 | 244944 | 1 | 1 | 67 | 29682 | 4 | 3 | 94 | HB1966 | 1 | 2 |
| 14 | 25915 | 3 | 3 | 41 | Hagere | 2 | 3 | 68 | 239077 | 1 | 3 | 95 | Wild Cross 01 | 1 | 1 |
| 15 | Mezezo | 2 | 3 | 42 | 27507 | 4 | 1 | 69 | 17190 | 3 | 2 | 96 | Wild Cross 02 | 1 | 1 |
| 16 | 26514 | 2 | 2 | 43 | 64265 | 1 | 3 | 70 | 15260 | 3 | 3 | 97 | Felamit | 3 | 2 |
| 17 | 242096 | 2 | 1 | 44 | 234305 | 3 | 3 | 71 | 243574 | 2 | 4 | 98 | Guta | 1 | 2 |
| 18 | 4496 | 1 | 2 | 45 | 242067 | 2 | 2 | 72 | Aruso | 1 | 2 | 99 | Debark-1 | 2 | 2 |
| 19 | 64255 | 4 | 1 | 46 | 237839 | 1 | 3 | 73 | 1637 | 4 | 2 | 100 | Muga | 1 | 2 |
| 20 | 235059 | 2 | 2 | 47 | 3489 | 2 | 2 | 74 | 243586 | 3 | 3 | 101 | Cross #41/98 | 1 | 2 |
| 21 | 29689 | 4 | 2 | 48 | 16861 | 4 | 4 | 75 | 17220 | 2 | 2 | 102 | Illala-01 | 3 | 2 |
| 22 | HB-1307 | 1 | 2 | 49 | 30230 | 2 | 4 | 76 | 30228 | 2 | 3 | 103 | Adoshe | 1 | 2 |
| 23 | 16862 | 4 | 4 | 50 | 17231 | 2 | 3 | 77 | 244890 | 1 | 3 | 104 | HB1965 | 1 | 2 |
| 24 | 17672 | 1 | 1 | 51 | 16863 | 1 | 4 | 78 | 1697 | 1 | 2 | 105 | Adena | 3 | 2 |
| 25 | Gobe | 1 | 2 | 52 | 240795 | 4 | 2 | 79 | 17685 | 1 | 3 |  |  |  |  |
| 26 | 29705 | 4 | 3 | 53 | Shege | 1 | 2 | 80 | 16737 | 4 | 3 |  |  |  |  |
| 27 | 244774 | 4 | 2 | 54 | 244771 | 4 | 2 | 81 | 3248 | 1 | 4 |  |  |  |  |

Regional Code: Oromia=1, Amhara=2, Tigray=3, SNNP=4, Altitudinal code: <2000=1, 2001-2500=2, 2501-3000=3, >3001=4, Pedigree: wild cross 01(SLB15-05/11 Spont 96-3/3 Roho//Alger/ceres 362-1-1/5/Roho/4/Zanbaka/3/ER/APm//Lignee 131) and wild cross 02 (Soufara-02/3/ RM 1508/ Por//W12269/4/HMl-02/Arabi Abiad//ER/APM/5/Sl-B15-05/4/H-Spont.96.3/3/Roho//Alger/Ceres 362-1-1) from Debre Zeit Agricultural Research Center. Numbers in the genotypes list are accessions while names are improved varieties and local checks/landraces.
