# Supplementary material for: Single nucleotide polymorphism (SNP) markers for genetic diversity and population structure study in Ethiopian barley (Hordeum vulgare L.) germplasm
Source: BMC Genom Data. 2023 Feb 14;24:7. doi: 10.1186/s12863-023-01109-6 (PMC9930229; doi:10.1186/s12863-023-01109-6)
Supplement: Supplementary file 2 — Additional file 2: Figure S1. The administrative map of Ethiopia indicates the collection points of the barley genotypes. Dots represent the barley genotypes in different colors depending on various region of origins and altitude ranges, according to the legend. [file 12863_2023_1109_MOESM2_ESM.docx]

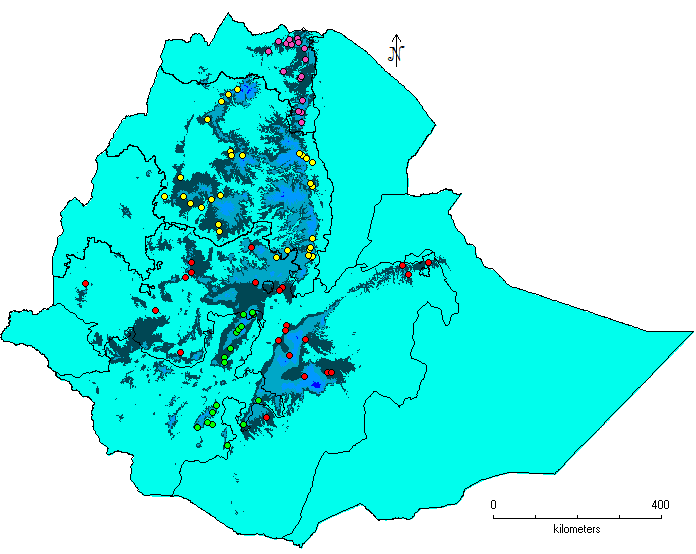

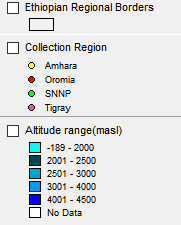


**Additional file 2: Figure S1**. The administrative map of Ethiopia indicates the collection points of the barley genotypes. Dots represent the barley genotypes in different colors depending on various region of origins and altitude ranges, according to the legend.
